# Applications of Artificial Intelligence in Nursing Care: A Systematic Review

**DOI:** 10.1155/2023/3219127

**Published:** 2023-07-26

**Authors:** Adrian Martinez-Ortigosa, Alejandro Martinez-Granados, Esther Gil-Hernández, Miguel Rodriguez-Arrastia, Carmen Ropero-Padilla, Pablo Roman

**Affiliations:** ^1^Emergency Department, San Cecilio University Hospital, Granada, Spain; ^2^Faculty of Health Sciences, Department of Nursing Science, Physiotherapy and Medicine, University of Almeria, Almeria, Spain; ^3^Torrecárdenas University Hospital, Andalucia, Spain; ^4^Research Group CTS-1114 Health Advances and Innovation, University of Almeria, Spain; ^5^Health Research Centre, University of Almeria, Almeria, Spain

## Abstract

**Aim:**

To synthesise the available evidence on the applicability of artificial intelligence in nursing care.

**Background:**

Artificial intelligence involves the replication of human cognitive abilities in machines, allowing to perform tasks that conventionally necessitate human cognition. However, its application in health sciences is a recent one, and its use is currently limited to supporting the diagnosis and prognosis of hospitalised patients, among others. *Evaluation*. A systematic review was conducted in the PubMed-Medline, Scopus, CINAHL, Web of Science, and Nursing & Allied Health databases until September 2022, following the PRISMA guidelines. *Key Issues*. A total of 21 articles were selected for the review. The different applications of artificial intelligence in nursing identified comprised (i) advances in early disease detection and clinical decision making; (ii) artificial intelligence-based support systems in nursing for patient monitoring and workflow optimisation; and (iii) artificial intelligence insights for nursing training and education.

**Conclusion:**

Artificial intelligence-based systems demonstrated increased autonomy of patients and professionals in care processes such as wound management through guided instructions, improved workflows, and efficiency in terms of time, materials, and human resources. *Implications for Nursing Management*. Artificial intelligence applied to nursing practice can be a very useful resource for professionals, managers, and supervisors. It has the potential to change current working flow systems and may serve as a down-to-earth resource to support nursing professionals in their decision-making process that ensures high quality and patient safety care.

## 1. Introduction

**Figure 1:**
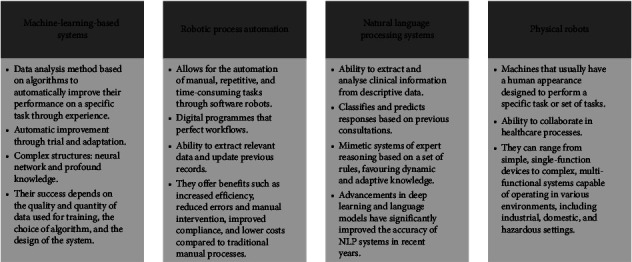
Types of artificial intelligence and their main characteristics in selected articles.

Artificial intelligence (AI) refers to the simulation of human intelligence in machines that are designed to perform tasks that typically require human cognition, such as problem solving, decision making, and pattern recognition [1, 2]. Due to its access to information, AI-based support systems can assist in making clinical decisions and therefore achieve better medical attention based on evidence [3–5]. In order to understand what AI entails, it is important to consider the various functions that contribute to its intelligence. These functions include machine learning (ML), natural language processing (NLP), behavioural pattern recognition, search engine capabilities, image and sound analysis, environmental perception, databases, information classification, and artificial neural networks. Additionally, another important aspect of AI is robotic process automation (RPA), which refers to the use of software robots to automate repetitive and routine tasks and physical robots, which are physical machines that can be programmed to perform tasks in the physical world [6, 7] (Figure 1).

The application of AI as a support system in healthcare has gained special relevance in recent decades, mainly as a result of growing data ecosystems in healthcare systems [8, 9]. In general terms, AI-based support systems offer a potential resource for reducing the cost of healthcare, increasing the efficiency of said services, and creating a highly valued support system for the well-being of patients and the healthcare sector in general, contributing to the satisfaction and clinical safety of patients and their family members [3–5]. However, the introduction of AI-based support systems in nursing care continues to cause concerns and debates due to the fear that this type of technology could eventually replace human interactions, jeopardising the ethics of care, and above all, that AI could eventually replace the functions of nurses [10]. Other ethical issues include the management of bias in data and its use to generate algorithms [11]. Despite the challenges, the use of AI in nursing aims to provide support and improve outcomes. As observed in the literature, the technology can address certain issues such as lack of expertise or inadequacy in experience, streamline documentation, and provide access to current evidence-based practices to ensure high-quality patient care, thus reducing the feeling of frustration that professionals have due to the organisational burden [10, 12]. Nurses play a crucial role in the delivery of patient care, and the increasing demand for high-quality, evidence-based practices has put pressure on the nursing workforce to stay up to date with the latest technological advancements [12]. In this regard, AI has the potential to support nursing practices by providing real-time decision support, reducing the time spent on administrative tasks, and facilitating the efficient management of patient data and care [13, 14].

In light of the rapidly evolving technological advancements in healthcare, it is important to evaluate the potential applications of AI on nursing practice. In this vein, recent systematic reviews have revealed promising results that AI can offer in the area of diagnosis and prognosis of certain clinical situations, such as cancer or new-onset pathologies in hospitalised patients [15, 16]. However, to our knowledge, the current body of literature that focuses on the application of different types of AI-based support systems in nursing is still limited, despite constituting one of the largest potential users of this type of technology as experts in caregiving. Thus, the aim of this review was to synthesise the available evidence on the applicability of artificial intelligence in nursing care.

## 2. Methods

### 2.1. Design

A systematic review was conducted in September 2022, following the recommendations of the Preferred Reporting Items for Systematic Reviews and Meta-Analyses (PRISMA) statements [17] (Supplementary File 1). The clinical question that responds to the aim of this review used a PCC structure (population-concept-context) [18] and is stated as follows: What is the applicability of artificial intelligence (C) in nursing (P) for patient care (C)? The review protocol was not registered.

### 2.2. Search Strategy

The databases that were consulted include PubMed-Medline, Scopus, CINAHL, Web of Science, and Nursing & Allied Health. In order to identify potential studies in the different databases, a search strategy was developed in collaboration with a research librarian and an information specialist that combined natural and structured language through the Medical Subject Headings (MeSH) (Table 1).

### 2.3. Selection Criteria

The inclusion criteria for the study were as follows: (i) original studies that focused on the use of AI in nursing and healthcare practice or (ii) nursing education, (iii) published in English or Spanish, and (iv) published until September 2022. Articles relating to nursing education were regarded as an integral part of nursing practice, as it furnishes the necessary knowledge and skills for providing quality patient care. On the other hand, (i) review studies, (ii) republications, (iii) editorials, and (iv) studies on animals were not considered.

### 2.4. Data Screening

The initial selection process in this study involved searching through the main health science databases (as listed in Table 1) to locate relevant evidence on the topic being addressed. This was followed by a two-stage screening process. In the first stage, two researchers (A.M.-O. and M.R.-A.) conducted an independent and parallel reading of the titles and abstracts of the available articles, taking into account the predefined selection criteria [19]. After this stage, duplicates were removed.

In the second stage, the selected articles underwent a more in-depth reading by the reviewers. To ensure methodological rigidity, a third auditor (P.R.) was consulted in case of any discrepancies, and a consensus on article eligibility was reached through rechecking the information [20]. Finally, the authors evaluated the methodological quality of the selected articles and classified them based on the type of AI used, the target population, and the purpose for which it was implemented [21].

### 2.5. Quality Evaluation

Appropriate methodological quality analysis tools were utilised for each study, using the Joanna Briggs Institute (JBI) Critical Appraisal Tools [22]. For articles with a mixed design, methodological quality was assessed with the Mixed Methods Appraisal Tools (MMAT) [23, 24].

### 2.6. Data Abstraction and Synthesis

The reviewers held discussions to determine the variables, as well as the nature and extent of the information to be extracted from the eligible articles, to ensure consistency and clarity of charted data [25]. After piloting on a sample of 5 articles, a custom data extraction was created in Microsoft Excel and iterated on. The most significant data of the selected studies included (i) author(s) and publication year, (ii) study design, (iii) participants and population, (iv) variable(s), (v) type of artificial intelligence, (vi) application in nursing practice, (vii) main findings, and (viii) mean level of compliance in the evaluation of methodological quality. Meta-analysis was not considered due to the heterogeneity of methodologies among the selected articles. Once the data were extracted, they were reviewed and discussed to deductively organise the main findings into clear and explicit categories in order to provide a critical synthesis of the cumulative evidence [26, 27].

## 3. Results

**Figure 2:**
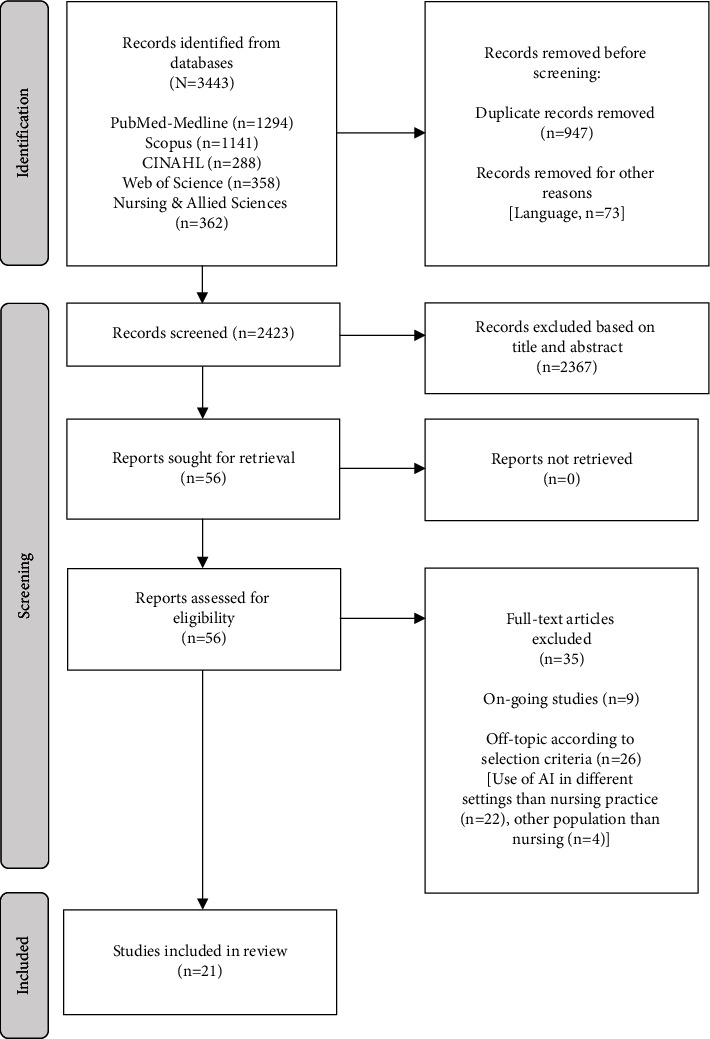
Flowchart depicting the article selection process.

The initial search yielded a total of 3,443 documents. After eliminating duplicates and selecting the articles by title and abstract, 56 full-text publications were reviewed according to the established selection criteria. In the end, a total of 21 articles were included in this review (Figure 2).

### 3.1. Included Studies

The synthesis of the included studies can be seen in Table 2. The sample size of the studies ranged from 10 participants to 230,936 participants, with a mean sample size of 14,948 participants. The predominant study population consisted of ambulatory patients (*n* = 10), healthcare professionals (*n* = 7), hospitalised patients (*n* = 5), students (*n* = 3), and caregivers (*n* = 1). Regarding the aim of the study, the different applications of artificial intelligence were differentiated as a support system for early disease detection (*n* = 4), clinical decision making (*n* = 3), patient monitoring (*n* = 7), workflow optimisation (*n* = 3), and nursing training and education (*n* = 3). In addition, some descriptive studies (*n* = 1) analysed the acceptance of the use of AI in the healthcare setting from a social and occupational perspective. Overall, 18 studies (85.7%) showed positive results in the application of this technology in nursing practice, while 3 studies (14.28%) indicated no impact on their results.

### 3.2. Methodological Quality: Assessment of Bias

The evaluation of the methodological quality of the articles included in this review showed a mean level of compliance of 74.60%. The interval risk of bias between the studies was 88.8% (*n* = 2) to 42.85% (*n* = 1) (Supplementary File 2) [22, 23].

The results of this review were presented based on the application of each type of AI discussed in the selected articles [28]. Thus, the categories used for the qualitative comprised the advancements in early disease detection and clinical nursing decision making, the utilisation of AI-based support systems for patient monitoring and workflow optimisation, and the use of AI for nursing training and education. The main AI-based systems studied include ML, RPA, NLP, and physical robots (Figure 1) [29–31].

### 3.3. Advances in Early Disease Detection and Clinical Decision Making

Of the selected studies, 33.33% (*n* = 7) applied AI technology based on ML models, of which 57.14% (*n* = 4) were used for the early disease detection. The diagnostic criteria of nurses and doctors improved by 12% and 10%, respectively, as a result of assistance from AI. This led to improved diagnosis of complex conditions, enhancing the accuracy and effectiveness of patient care. Likewise, the professionals who used AI reported an increase in their confidence in evaluations and clinical decision making by providing real-time analysis and interpretation of patient data and a decrease in biopsy requests, highlighting the improvement in the dermatological field and in the detection of certain cardiac diseases, such as cardiac amyloidosis [31, 32]. In the same manner, the research by Ginestra and collaborators [33] and Sandhu and collaborators [34] used different AI-based support systems for early warnings of sepsis risk through static (demographics and previous illnesses) and dynamic (vital signs and laboratory data) patient characteristics. Conversely, Horng and collaborators [35] used a ML system combined with NLP to assess the ability to diagnose patients by adding comments that doctors and nurses wrote in medical records. Thus, the specificity, sensitivity, and area under the curve for the detection of sepsis were higher in the model that used the comments from medical records than those intelligent systems that only evaluated static and dynamic characteristics.

Finally, one of the main measures used in the studies to assess the effect of AI on clinical nursing practice was the use of reference diagnoses. [31, 32], in which at least three certified physicians independently reviewed each case. These diagnoses were obtained through a collective intelligence approach, in which the different differential diagnoses of each physician were ranked through a voting system to finally obtain a principal or reference diagnosis, in order to assess the effect produced by the AI in each case [7, 32, 36]. On the other hand, satisfaction surveys were used as a method of measuring the effect of AI on nurses, patients, and students [30, 36, 37].

### 3.4. AI-Based Support Systems in Nursing for Patient Monitoring and Workflow Optimisation

Poncette and collaborators [38] demonstrated in their results that 93% of participants from intensive care units (ICUs) were in favour of the use of AI-based support systems, specifically the use of wireless sensors to reduce false positive alarms. In regard to predicting the risk of readmission in these units, the conventional scales used obtained significantly lower sensitivity and area under the curve, compared with models that used ML algorithms [39]. In 4.76% (*n* = 1) of the studies included in this review, programmes that integrated image recognition were also analysed to extract data and update the medical records of patients [40]. Specifically, programmes carried out to manage diabetic foot ulcers were evaluated by three different devices and their effectiveness was compared with an expert wound care nurse, demonstrating excellent intra-reliability and inter-reliability in their results with a value greater than 0.9 in length, width, and wound area.

Regarding the use of a conversational agent or chatbots, authors such as da Silva Lima Roque et al. [41] examined its use for examining and identifying personalised wound treatments. The content validity of this resource was rated as excellent by healthcare experts in wounds, and the images shown to the users were comprehensible in 88% of the cases. Among the participants, the most repeated assessments of the AI-based support system were easy to use, suitable for any user, dynamic, entertaining, and quick to respond. However, they also pointed out negative aspects such as a lack of realism or failures to capture long sentences [29, 36]. Lastly, another AI modality, rule-based expert systems, was studied in 4.76% (*n* = 1) of the studies, with the aim of telemonitoring patients with known atrial fibrillation [42]. These intelligent systems were programmed with established guidelines recognised in a clinical practice guide with personalised recommendations for each patient, improving adherence to treatment, availability of clinical data in real time, and its control from the medical community. Likewise, another resource used was telephone follow-up for postsurgical patients [43], comparing the call time and the feedback received between an AI system and a nurse. The call time was shorter in the intelligent system (1-2 min) and with better feedback when compared to that of the nurse (3–6 min) (*p* < 0.01), though the effectiveness of the follow-up was the same in both situations.

Of all the articles analysed, 28.57% (*n* = 6) used robots with AI-based technology for various uses in patient monitoring and workflow optimisation, including rehabilitation and respiratory exercises [44], evaluation of the elderly and accompaniment of people with dementia [45, 46], and behavioural therapy [47]. Likewise, the characteristics and aspects to consider in the design and inclusion of robots in the healthcare field were examined [48, 49]. Using robots for rehabilitation and respiratory exercises in patients with COPD has been shown to be effective in reducing hospital readmissions, the workload of nursing professionals, and visits to health centres or surgeries [44]. On the other hand, in other studies that used these devices to study frailty in the elderly [45, 46] or in behavioural therapy to relieve pain in subcutaneous punctures in infants [47], no differences were observed in the average time of execution or precision and no statistically significant differences were observed in pain control. However, it did prove to be an effective way to distract children.

### 3.5. AI Insights for Nursing Training and Education

There is a growing interest exploring the potential of AI-based support systems to improve nursing training and education. A recent study by Chang [37] investigated the use of AI-powered simulation to enhance nursing clinical decision-making skills. The results showed that students who underwent AI-enhanced simulation training performed significantly better on clinical decision-making assessments compared to those who received traditional simulation training. Chatbots were used with similar results to support nursing training about vaccines for pregnant women, showing significantly better results (*p* < 001) regarding the expertise and level of knowledge on the subject, thereby improving the care and safety received by the patient [36, 37]. Similarly, other studies concluded that AI-based educational resources have the potential to increase engagement and enhance their learning outcomes in nursing education. These findings suggest that AI has the potential to play an important role in improving nursing training and education [29, 36].

## 4. Discussion

The aim of this review was to synthesise the available evidence on the applicability of artificial intelligence in nursing care. After analysing the selected studies, contrary to other studies on this topic, our findings suggest that the application of AI in the nursing field could improve care delivery, contingent on the type of artificial intelligence used and its various applications, such as early diagnosis, clinical nursing decision making, patient care management and monitoring, workflow optimisation, and nursing education [29, 41, 50].

Among the uses analysed in this review, similar to other recent reviews, one of the most commonly used types of support systems was based on ML models for diagnosis [15, 33–35, 51]. One possible explanation for the predominant use of this type of AI may be due to the use of its learning system for activities that are traditionally carried out by humans, but with much less time and expense [52]. These technological advances in AI are currently and predominantly focused on diagnosing diseases such as Alzheimer's, cancer, chronic or dermatological diseases, and more. This allows health professionals to have more time with their patients, use a holistic approach, and even improve patient satisfaction with health organisations [14, 15, 32, 40]. Therefore, diagnostic reliability should be considered in these studies, increasing the presence of comorbidities with the possibility of combining it with NLP models to improve the interpretation of comments from clinical histories [31, 33–35].

One of the types of artificial intelligence that has perhaps received the most attention in recent years within NLP is the rule-based expert system, not only for managing cases of COVID-19 symptoms, but also providing care for patients with social anxiety and older adults who are immunocompromised or at risk of isolation [46, 53]. These types of systems have proven to be effective in monitoring patients remotely in real time, improving the availability of updated clinical data, allowing more time for nursing care, and improving patient safety in primary care, special services, and remote areas [39, 42, 44, 54]. However, like other types of AI, the majority of systems that are based on monitoring patients from a distance still require longer learning phases to achieve clinical reliability, which impedes its implementation [54]. On the other hand, the use of other types of AI, such as the automation of robotic processes or physical robots [45–47], has been shown to be satisfactory in some studies through their use as a mobile application, by increasing the effectiveness and efficiency of nurses' work in the field of tissue injuries and optimising the identification and management of wounds, pain control during venepuncture, or adherence to treatment, among others [29, 55]. Nonetheless, there is still little known about these types of AI, unlike other more widespread ones such as systems based on natural language processing or explainable artificial intelligence [56].

The conversational agents developed through dialogue systems, NLP, and statistical models have demonstrated positive results in reducing the workload of nursing professionals in hospital administrative tasks [57], treatment and follow-up of wound management [41], triage and nursing diagnosis [11, 30], and the training of future professionals [29, 58]. Despite the growing amount of evidence on this type of AI, other authors point out the shortcomings that this type of technology still has, including poor voice recognition, lack of expression and emphasis in speech, and frequently interrupted conversation, all of which prevent results with clinical relevance greater than usual practices from being reached [36, 43, 50]. Having said that, there are a growing number of relevant organisations, such as the Nursing and Artificial Intelligence Leadership Collaborative group, as well as important tech-companies, such as OpenAI, that are currently thriving to identify priority areas for action, opportunities, and recommendations to address these concerns in healthcare practice [59–61].

However, there are some limitations to this study that should be noted. First, AI applied in healthcare and nursing care is still a growing practice with limited evidence due to studies which are in development and have a great heterogeneity in AI types and settings. Therefore, generalisations of the proposed results should be made cautiously. On the other hand, only the term “artificial intelligence” was used as a keyword to describe all AI methods. Although the MeSH term “artificial intelligence” includes most of AI-based systems in its tree structures, some features utilising specific AI methods but are not explicitly labelled as AI may have been overlooked. Carrying out a meta-analysis or meta-regression was not considered in this review due to the heterogeneity in the types of AI, populations, and study variables. In general, this review contributes to the existing knowledge of applying AI-based systems in the healthcare field and nursing practice. Although positive results were shown for most of the analysed types, a greater number of studies are needed to consider the current limitations of these systems and the needs of professionals-users for the development of AI-based systems. There is still abundant room for further progress with these systems in terms of guaranteeing not only professional autonomy but also improved access to health information sources in order to optimise their use in multitasking to cover the greatest number of variables that may affect the patient, environment, clinical practice, and different medical services. Further research is needed to investigate how previous research findings using AI-based systems with virtual reality or simulated scenarios can be implemented in real-life clinical nursing practice or analyse how these AI-based support systems may improve patient safety and assist nurses in particular clinical settings.

### 4.1. Implications for Nursing Management

AI applied to nursing practice can be a down-to-earth resource for professionals, managers, or supervisors, with positive results in patient care and safety. It has the potential to change current working flow systems and provides support to nursing professionals when making decisions. AI-based systems are flexible tools which can adopt various essential functions in nursing care, such as guiding the patient with personalised instructions or remotely monitoring the patient in real time. Furthermore, it can be used in community care, remote areas, or in the hospital setting by identifying the possible diagnosis of the user early on, thereby accelerating the healthcare process. The greatest challenge in the development and implementation of this type of technology, however, continues to be the involvement and active participation of healthcare professionals and their commitment to its use.

## 5. Conclusions

This review offers a compilation of the available evidence on the different applications of AI-based support systems that can be implemented in nursing practice. The systems which are based on machine learning and natural language processing are the most widely used, demonstrating better results in different healthcare processes. Despite the limitations that still exist with this type of technology, the results of the different types of AI are promising. AI-based systems can assist in early diagnosis, clinical decision making, patient monitoring, and workflow optimisation. However, it is important to consider ethical and privacy concerns as well as ensure that AI is used to augment and enhance the role of nurses rather than replace them [61].

## Figures and Tables

**Table 1:** Search strategies used in each database.

Database	Search strategy
PubMed-Medline	((Artificial intelligence[Title/Abstract]) OR (artificial intelligence[MeSH terms])) AND ((((nurse[Title/Abstract]) OR (nurses[Title/Abstract])) OR (nursing[Title/Abstract])) OR (nursing[MeSH terms]))
Scopus	TITLE-ABS-KEY (“nurses”) OR TITLE-ABS-KEY (“nurse”) OR TITLE-ABS-KEY (“nursing”) OR INDEXTERMS (“nursing”) AND TITLE-ABS-KEY (“artificial intelligence”) OR INDEXTERMS (“artificial intelligence”)
CINAHL	(((((MH “nursing”) OR (TI “nursing”) OR (AB “nursing”)) OR ((TI “nurses”) OR (AB “nurses”)) OR ((TI “nurse”) OR (AB “nurse”)))) AND ((TI “artificial intelligence”) OR (AB “artificial intelligence”) OR (MH “artificial intelligence”))
Web of Science	((((((((TI=(nurse)) OR AB=(nurse)) OR TI=(nurses)) OR AB=(nurses)) OR TI=(nursing)) OR AB=(nursing)))) AND ((TI=(artificial intelligence)) OR AB=(artificial intelligence))
Nursing & Allied Health	(MESH(nursing) OR AB(nursing) OR TI(nursing) OR AB(nurse) OR TI(nurse) OR AB(nurses) OR TI(nurses)) AND (MESH(artificial intelligence) OR AB(artificial intelligence) OR TI(artificial intelligence))

**Table 2:** Overview of selected articles.

Authors (year)	Design	Participants (*n*) and population	Variable (s)	Type of AI	Application in nursing	Main findings	MLC (%)
Dinesen et al. (2022)	Exploratory study	39 patients with dementia	Perceptions about the response in the mood of the patients	Physical robot	Social work and accompaniment	The patient's mood improved, making them feel calmer and improving the interaction with the subject	88.88
Chang (2022)	Quasi-experimental study	43 postgraduate nursing students	Teaching intervention with virtual reality and learning achievements	NLP	Nursing education	The most valued positive aspects were the availability of a safe environment, the possibility of multiple attempts, overcoming difficulties, the consolidation of knowledge, and professional empowerment	88.88
Cho et al. (2022)	Prospective intervention study	19 triage nurses	Time, completion rate of records, and accuracy	NLP	Registration and classification of patients by voice	Improved patient registration and classification time, requiring a technical complement for improved sensitivity and accuracy	81.81
Chan et al. (2022)	Cross-sectional prospective descriptive study	28 ambulatory and hospitalised patients	Wound length, width and area, device reliability	Robotic process automation	Wound treatment	Excellent reliability. Useful for monitoring diabetic foot ulcers with a statistically significant difference compared to traditional measures.	87.5
Jain et al. (2021)	Case series study	40 doctors and nurses	Agreement with dermatologists and confidence in the diagnosis	Machine learning	Diagnosis and decision making	Improved nursing diagnosis by 10% and 12%, respectively	80.0
Lima-Roque et al. (2021)	Descriptive study	17 ambulatory patients, main caregivers, and nurses	Content validity (identify characteristics of the wound and healing process). Participant satisfaction.	NLP	Wound treatment	Excellent content validity. A great help for wound management and easy to use. Adapted to the general population.	50.0
Chang et al. (2021)	Quasi-experimental study	36 nursing students	Learning, student confidence in the course, and user satisfaction	NLP	Nursing education	The chatbot improved student learning compared to traditional teaching techniques with statistically significant results	77.7
García-García et al. (2021)	Retrospective cross-sectional descriptive study	11,586 ambulatory and hospitalised patients	System sensitivity, specificity, and precision with AI	Machine learning	Diagnosis and decision making	Able to better identify cases of cardiac amyloidosis when the sample contained many patients with heart failure. No external validity.	87.5
Hong et al. (2021)	Randomised clinical trial	447 ambulatory patients with COPD	Quality of life, hospital stay, admissions for exacerbations, and visits to the doctor. Patient satisfaction.	Physical robot	Remote monitoring	Remote monitoring alerted healthcare providers to COPD complications. The exercises carried out with the robot reduced hospital admissions and improved the quality of life of patients.	53.8
Jang et al. (2021)	Descriptive study	117 doctors and nurses	Perceptions about the necessary functions of the robot	Physical robot	Remote monitoring	The most requested functions in the surveys were measuring vital signs, obstacle detection, and the alarm system, among others	62.5
Bian et al. (2020)	Mixed exploratory study	270 outpatients	On-call time, feedback, and type of patient feedback	NLP	Remote monitoring	The nurse received less feedback than the AI. The on-call time was similar in both. The AI tracked 5–7 patients at a time.	71.4
Poncette et al. (2020)	Descriptive study	86 ICU nurses and doctors	Opinions and perceptions of AI to improve work in the ICU	Machine learning	Remote monitoring	93% were in favour of using wireless sensors and reducing false positive alarms. Refusal to use a smart watch and augmented reality glasses for patient monitoring.	62.5
Shorey et al. (2020)	Qualitative study	30 nursing students and clinical professors	Advantages and disadvantages of virtual reality in learning communication skills	NLP	Nursing education	The most cited positive aspects were the availability of multiple attempts, a comfortable and safe environment, and instant feedback from the virtual patient	80.0
Lee et al. (2020)	Mixed study	198 hospitalised and healthcare centre patients	Perceptions about the necessary functions of the robot	Physical robot	Remote monitoring	Some of the advantages cited were access to clinical reports, communication with health professionals, and physical and emotional support	85.7
Sandhu et al. (2020)	Qualitative study	15 nurses and emergency room doctors	Perceptions and experiences about the usefulness of the application and considerations when implementing AI	Machine learning	Diagnosis and decision making	Triage nurses had a positive impression of sepsis control, while clinicians suggested that they would be more confident in the model if it had fewer false positives	80.0
Ginestra et al. (2019)	Descriptive observational study	287 doctors and nurses	Usefulness of the alert, impact on care	Machine learning	Diagnosis and decision making	Despite the excellent predictive characteristics of the EAS, new clinical information or changes in treatment were rarely reported	87.5
Boumans et al. (2019)	Randomised clinical trial (pilot)	42 outpatients	Time to complete the interview. Fragility, well-being, and resilience index. Acceptance of the robot.	Physical robot	Diagnosis	The questionnaires were completed faster by the nurse than the robot. Resilience index data were more accurate in the nurse than in the robot.	76.9
Rojas et al. (2018)	Retrospective cohort study	24,885 hospitalised patients in the ICU	Sensitivity, specificity, and ABC of the AI system	Machine learning	Remote monitoring	The GBM obtained significantly higher sensitivity and ABC (*p* < 0.001) than the SWIFT and MEWS scales	63.6
Jibb et al. (2018)	Randomised clinical trial (pilot)	40 oncology Paediatric ambulatory	Pain and fear with FPS and BAAS scales. Time needed to complete interaction. Acceptance of the robot.	Physical robot	Management of pain and fear	The control group with a distracting robot felt more relaxed than the intervention group with a conductive therapy robot. Both managed to relieve pain and stress.	84.6
Horng et al. (2017)	Retrospective cohort study	230, 936 emergency room patients	Sensitivity, specificity, and ABC of the AI system	Machine learning	Diagnosis and decision making	The use of vital signs together with freely formulated text improved the criteria for an infectious diagnosis, increasing the sensitivity and specificity of the system. The ABC was higher in the model that evaluated all of the variables.	72.7
Parimbelli et al. (2016)	Mixed study (pilot)	10 ambulatory patients with AF	Compliance with prescribed treatment	NLP	Remote monitoring	MobiGuide increased treatment adherence for AF patients and the availability of up-to-date clinical data. Interdisciplinary work was improved.	42.8

ABC: area below curve; BAAS: behavioural approach-avoidance scale; COPD: chronic obstructive pulmonary disease; EAS: early alert system; AF: atrial fibrillation; FPS: face pain scale; GBM: gradient boosting machine; AI; artificial intelligence; MEWS: modified early warning score; MLC: mean level of compliance; NLP: natural language processing; SWIFT: stability and workload index for transfer; ICU: intensive care unit.

## Data Availability

Data sharing is not applicable to this article as no new data were created or analysed in this study.

## Supplementary Materials

Supplementary Materials Supplementary File 1: Preferred Reporting Items for Systematic Reviews and Meta-Analyses (PRISMA) checklist of our systematic review report. Supplementary File 2: risk of bias summary using JBI Critical Appraisal Tools and Mixed Methods Appraisal Tools for included studies.
