# Supplementary material for: Applications of Artificial Intelligence in Nursing Care: A Systematic Review
Source: J Nurs Manag. 2023 Jul 26;2023:3219127. doi: 10.1155/2023/3219127 (PMC11919018; doi:10.1155/2023/3219127)
Supplement: Supplementary Materials — Supplementary File 1: Preferred Reporting Items for Systematic Reviews and Meta-Analyses (PRISMA) checklist of our systematic review report. Supplementary File 2: risk of bias summary using JBI Critical Appraisal Tools and Mixed Methods Appraisal Tools for included studies. [file 3219127.f1.zip › Supplementary_File_2_R2.docx]

SUPPLEMENTARY FILE 2

Suppl. Table 1: Risk of bias summary using JBI Critical Appraisal Tools and Mixed Methods Appraisal Tools for included studies

| Authors (year) | Item 1 | Item 2 | Item 3 | Item 4 | Item 5 | Item 6 | Item 7 | Item 8 | Item 9 | Item 10 | Item 11 | Item 12 | Item 13 | Assessment tool (MLC) |
| --- | --- | --- | --- | --- | --- | --- | --- | --- | --- | --- | --- | --- | --- | --- |
| Dinesen et al. (2022) |  |  |  |  |  |  |  |  |  |  |  |  |  | JBI (88.88%) |
| Chang (2022) |  |  |  |  |  |  |  |  |  |  |  |  |  | JBI (88.88%) |
| Cho et al. (2022) |  |  |  |  |  |  |  |  |  |  |  |  |  | JBI (81.81) |
| Chan et al. (2022) |  |  |  |  |  |  |  |  |  |  |  |  |  | JBI (87.5%) |
| Jain et al. (2021) |  |  |  |  |  |  |  |  |  |  |  |  |  | JBI (80%) |
| Lima-Roque et al. (2021) |  |  |  |  |  |  |  |  |  |  |  |  |  | JBI (50%) |
| Chang et al. (2021) |  |  |  |  |  |  |  |  |  |  |  |  |  | JBI (77.77%) |
| García-García et al. (2021) |  |  |  |  |  |  |  |  |  |  |  |  |  | JBI (87.5%) |
| Hong et al. (2021) |  |  |  |  |  |  |  |  |  |  |  |  |  | JBI (53.84%) |
| Jang et al. (2021) |  |  |  |  |  |  |  |  |  |  |  |  |  | JBI (62.5%) |
| Bian et al. (2020) |  |  |  |  |  |  |  |  |  |  |  |  |  | JBI (71.42%) |
| Poncette et al. (2020) |  |  |  |  |  |  |  |  |  |  |  |  |  | JBI (62.5%) |
| Shorey et al. (2020) |  |  |  |  |  |  |  |  |  |  |  |  |  | JBI (80%) |
| Lee et al. (2020) |  |  |  |  |  |  |  |  |  |  |  |  |  | MATT (85.71%) |
| Sandhu et al. (2020) |  |  |  |  |  |  |  |  |  |  |  |  |  | JBI (80%) |
| Ginestra et al. (2019) |  |  |  |  |  |  |  |  |  |  |  |  |  | JBI (87.5%) |
| Boumans et al. (2019) |  |  |  |  |  |  |  |  |  |  |  |  |  | JBI (76.92%) |
| Rojas et al. (2018) |  |  |  |  |  |  |  |  |  |  |  |  |  | JBI (63.63%) |
| Jibb et al. (2018) |  |  |  |  |  |  |  |  |  |  |  |  |  | JBI (84.61%) |
| Horng et al. (2017) |  |  |  |  |  |  |  |  |  |  |  |  |  | JBI (72.72%) |
| Parimbelli et al. (2016) |  |  |  |  |  |  |  |  |  |  |  |  |  | MATT (42.85%) |

JBI: Joanna Briggs Critical Appraisal Tools; MATT: Mixed Methods Appraisal Tools; MLC: Mean level of compliance
